# Antianemia Drug Roxadustat (FG-4592) Protects Against Doxorubicin-Induced Cardiotoxicity by Targeting Antiapoptotic and Antioxidative Pathways

**DOI:** 10.3389/fphar.2020.01191

**Published:** 2020-08-05

**Authors:** Guangfeng Long, Hongbing Chen, Mengying Wu, Yuanyuan Li, Ling Gao, Songming Huang, Yue Zhang, Zhanjun Jia, Weiwei Xia

**Affiliations:** ^1^ Department of Clinical Laboratory, Children’s Hospital of Nanjing Medical University, Nanjing, China; ^2^ Nanjing Key Laboratory of Pediatrics, Children’s Hospital of Nanjing Medical University, Nanjing, China; ^3^ Department of Nephrology, Children’s Hospital of Nanjing Medical University, Nanjing, China; ^4^ Jiangsu Key Laboratory of Pediatrics, Nanjing Medical University, Nanjing, China

**Keywords:** Roxadustat, doxorubicin cardiotoxicity, HIF-1α, apoptosis, oxidative stress

## Abstract

Doxorubicin (DOX) is broadly used in treating various malignant tumors. However, its cardiotoxicity limits its clinical use. Roxadustat (FG-4592) is a new hypoxia-inducible factor prolyl hydroxylase (HIF-PHD) inhibitor and has been approved for treating anemia in chronic kidney diseases (CKD) patients. However, the role of FG-4592 in DOX-induced cardiotoxicity remains unknown. In this study, mouse cardiac function was evaluated by echocardiography, plasma LDH/CK-MB, and heart HE staining. Cell viability, apoptosis, oxidative stress, inflammation, and HIF-target genes were evaluated in mouse cardiac tissue and cardiac cells exposed to DOX with FG-4592 pretreatment. DOX-sensitive HepG2 and MCF-7 cell lines were used to evaluate FG-4592 effect on the anticancer activity of DOX. We found that FG-4592 alleviated DOX-induced cardiotoxicity shown by the protection against cardiac dysfunction, cardiac apoptosis, and oxidative stress without the effect on inflammatory response. FG-4592 alone did not change the cardiac function, cardiomyocyte morphology, oxidative stress, and inflammation *in vivo*. FG-4592 could protect cardiomyocytes against DOX-induced apoptosis and ROS production in line with the upregulation of HIF-1α and its target genes of Bcl-2 and SOD2. Importantly, FG-4592 displayed anticancer property in cancer cells treated with or without DOX. These findings highlighted the protective effect of FG-4592 on DOX-induced cardiotoxicity possibly through upregulating HIF-1α and its target genes antagonizing apoptosis and oxidative stress.

## Introduction

Doxorubicin (DOX) is an effective chemotherapeutic agent which is used in diverse cancers. However, the clinical application of DOX is severely restricted by its chemotoxicity, especially the cardiotoxicity (Simunek et al., 2009). Left ventricular dysfunction and heart failure were the most severe symptoms of DOX-induced cardiotoxicity (Pacher et al., 2002; Pacher et al., 2003). Although the potential pathogenic mechanisms of DOX-induced cardiotoxicity including increased production of reactive oxygen species (ROS), cell apoptosis, mitochondrial damage, and impaired calcium homeostasis have been investigated, the exact mechanism is still elusive (Kim et al., 2006; Li et al., 2006; Danz et al., 2009; Xu et al., 2012; Yin et al., 2018). Dexrazoxane is the only drug licensed by the US Food and Drug Administration for protecting heart against the DOX-induced cardiotoxicity under both experimental and clinical condition (Wouters et al., 2005; Lipshultz et al., 2010). But several studies found that dexrazoxane may interfere with the anticancer activity of DOX and also potentiate the myelosuppressive effects of DOX (Hasinoff, 2008). Therefore, it is important and urgent to find a new agent for treating DOX-induced cardiotoxicity.

Hypoxia inducible factor-1 (HIF-1) consists α and β subunits, and acts as an important transcription factor in the maintenance of oxygen homeostasis signaling system (Wang et al., 1995). In normal oxygen state, HIF-1 is unstable and rapidly degraded by hypoxia-inducible factor prolyl hydroxylase (HIF-PHD). PHD activity is inhibited when hypoxia occurs, and HIF-1 is translocated into the nucleus to activate the expression of target gene by binding hypoxia response element (HRE) sequence of the target gene promoter. More than 100 target genes of HIF-1 have been identified in human (Schodel et al., 2013). Increasing studies have found that HIF-1α plays a protective role in atherosclerosis, heart failure and ischemic heart diseases (Hewitson et al., 2004; Semenza, 2014; Sousa Fialho et al., 2019). In the DOX-induced H9c2 cell injury model, HIF-1a showed a protective effect by reducing oxidative stress and cell apoptosis (Spagnuolo et al., 2011). However, HIF-1 is easily degraded by HIF-PHD under normal oxygen condition. Therefore, it would be of great use to find a pharmacologic inhibitor of HIF-PHD for the treatment of DOX-induced cardiotoxicity.

Roxadustat (FG-4592/ASP1517) is a novel, orally administered agent that is a new potent HIF-PHD inhibitor developed by FibroGen. Roxadustat (Ai Rui Zhuo^®^ in China) has obtained its first approval in China for treating anemia in chronic kidney disease patients undergoing dialysis on 17 December 2018 (Dhillon, 2019). Roxadustat stabilizes HIF, reduces HIF-1α degradation and promotes HIF transcriptional activity by inhibiting the activity of HIF-PHD enzyme. Stabilization of the HIF induces the endogenous increase of target genes EPO (Semenza, 2000). HIFs also play a critical role in iron metabolism by targeting iron-related proteins, such as ferroportin 1 (FPN1), and transferrin receptor (TFR) (Xu et al., 2017). Previous studies have shown that FG-4592 increased HIF-1α expression and attenuated apoptosis induced by MPP+ in SH-SY5Y cells (Li et al., 2018). FG-4592 pre-treatment reversed the increment of Bax and the decrement of Bcl-2 induced by MPP+ partially. Moreover, mitochondrial biogenesis and oxidative respiratory response were increased by FG-4592 in dopaminergic neurons by regulating mitochondrial membrane potential (MMP), mitochondrial oxygen consumption rate (OCR) and ATP production (Li et al., 2018). Meanwhile, FG-4592 was also illustrated to counterbalance the oxidative stress by increasing the expression of HIF-1α target gene superoxide dismutase 2 (SOD2), nuclear factor erythroid 2-related factor 2 (Nrf-2), and heme oxygenase-1 (HO-1) which mediate ROS detoxification (Li et al., 2018). In addition, one recent study showed FG-4592 ameliorated the cisplatin-induced acute kidney injury through regulating apoptosis and inflammation (Yang et al., 2018). FG-4592 also had protective effects against atherosclerosis and high glucose-induced glomerular endothelial cells injury through upregulating HIF (Xie et al., 2019; Zhang et al., 2019). For cancer cells, HIFs increases the proliferation and metastasis of cancer cells by regulating the expression of some target genes (Keith et al., 2011). HIF inhibitors are currently undergoing the clinical evaluation as anticancer drugs. However, recent studies showed that FG-4592 could inhibit tumor growth of macrophage-abundant tumors (Nishide et al., 2019), and reduced viability and proliferation of human ovarian clear cell carcinoma ES2 cells (Price et al., 2019), suggesting an antitumor potential of FG-4592. In the present study, the role and potential mechanisms of FG-4592 in DOX-induced cardiotoxicity were investigated.

## Materials and Methods

### Antibodies and Agents

HIF-1α antibody (No10006421, 1:200) and TBARS (MDA) Assay Kit (No 10009055) were bought from Cayman chemical (Ann Arbor, MI, United States). Antibodies of Bax (No 2772) was purchased from Cell Signaling Technology (Danvers, MA, United States). Anti-SOD2 antibody (No ab13533) was provided by Abcam (Cambridge, United Kingdom). Bcl-2(No 26593-1-AP) and β-actin (No 60008-1-Ig) were bought from Proteintech, (Rosemont, IL, United States). Doxorubicin (No D1515) was purchased from sigma (Saint Louis, MO, United States). FG-4592 (No S1007) was from Selleck Chemicals IIc (Houston, TX, United States). MitoSOX™ Red Mitochondrial Superoxide Indicator (No M36008) was purchased from ThermoFisher Scientific (Waltham, MA, United States). Cell Counting Kit-8 assay kit (No KGA317) was from KeyGen Biotech (Nanjing, China). TNF-α (No DKW12-2720-096) and IL-6 (No DKW12-2060-096) ELISA kits were purchased from Dakewe Biotech (Shenzhen, China). Apoptosis detection kits (No 556547 and No 559763) were from BD Biosciences (San Diego, CA, United States). TUNEL BrightGreen Apoptosis Detection Kit (No A112-01) was bought from Vazyme Biotech (Nanjing, China).

### Animals and Treatment

Wild-type C57BL/6 mice (10–12 weeks old, weighing 25–28 g) were purchased from Model Animal Research Center of Nanjing University (Nanjing, China). All mice were housed in a specific pathogen-free condition with 12/12 h light/dark cycle and free access to food and water. To evaluate the role of FG-4592 in DOX-induced myocardial injury, mice were randomly divided into three groups as follows: control group (control group, n = 7), DOX-induced myocardial injury group (DOX group, n = 9), and DOX-induced myocardial injury with FG-4592 pre-treatment group (FG-4592+DOX group, n = 7). DOX (Sigma Aldrich, 15663-27-1) was administered to both DOX and FG-4592+DOX mice by a single intraperitoneal injection (12.5 mg/kg). Control mice received an equal volume sterile phosphate buffer saline (PBS) by intraperitoneal injection. The FG-4592 (Selleck Chemicals, S1007) was dissolved in DMSO at the concentration of 50 mg/ml and further diluted in PBS to 1 mg/ml. The mice were pretreated with FG-4592 for 48 h in FG-4592+DOX group at a dose of 10 mg/kg/day before DOX treatment. Besides, in order to assess the potential side effect of FG-4592 alone on heart, we performed another animal experiment by treating mice with FG-4592 or vehicle for 9 days (n = 7 in each group). After DOX administration for 7 days, cardiac function was evaluated by echocardiograms, then the mice were sacrificed. Blood samples were centrifugated for serum and stored at −80°C for further analysis. Left ventricular tissues were harvested to fix in 4% paraformaldehyde (PFA) for histology and the remaining tissues were frozen in liquid nitrogen for mRNA and protein analysis. All animal study protocols were approved by the Institutional Animal Care and Use Committee of Nanjing Medical University. (Nanjing, China). All animal work was performed at Animal Research Center of Nanjing Medical University.

### Echocardiography

Seven days after the injection of DOX, mice were anesthetized with isoflurane to measure the cardiac function when the heart rate stabilized at 400 to 500 beats per minute. The cardiac function was measured by a Vevo 2100 high-resolution *in vivo* imaging system (VisualSonics, Toronto, ON, Canada).

### Plasma Biochemical Analysis

Blood sample was taken from the inferior vena cava with heparin sodium-treated syringe and centrifuged at 3000 rpm for 20 min to collect the plasma. The levels of Creatine kinase-MB (CK-MB) and Lactate dehydrogenase (LDH) in plasma were enzymatically measured using an automatic biochemical analyzer.

### TBARS (MDA) Assay

To analyze the oxidative stress, we detected the MDA levels in heart left ventricular tissues and cultured H9c2 cells by using commercial TBARS assay kit following the manufacturer’s instruction. The data were measured by a microplate reader at 530–540 nm.

### Determination of Mitochondrial Superoxide Production

The generation of mitochondrial ROS was measured by flow cytometry (BD FACScan flow cytometer, Franklin Lakes, NJ, United States). Briefly, H9c2 cells were pretreated with FG-4592 for 24 h and then administrated with DOX for another 24 h. After that, the cells were incubated with 10 μM MitoSOX™ Red Mitochondrial Superoxide Indicator for another 20 min following the manufacturer’s instruction. The cells were collected and analyzed using flow cytometry. The mean fluorescent intensity was measured by using FlowJo software.

### Primary Culture of Neonatal Rat Ventricular Cardiomyocytes (NRVMs)

Primary cardiomyocytes were isolated from 2 days old neonatal Sprague-Dawley rats. Briefly, hearts from neonatal rats were removed and transferred into plates with precooling PBS. After removing atria, ventricles were digested with 0.25% trypsin for 30 min. The cells were harvested by centrifugation at 1000 × rpm for 5 min and plated in culture dishes containing medium (DMEM with10% Fetal Bovine Serum) in incubator for 2 h to remove nonmyocytes. The suspended cells were transferred to 96 plate. After the NRVMs were cultured for 2 days, cells were administrated with FG-4592 (1, 2.5, 5 μM) for 48 h or pretreated with FG-4592 (1, 2.5 μM) for 24 h followed by another 24 h treatment of DOX. Then cell viability assay was performed.

### Cell Culture and Treatment

The rat heart-derived cardiac cells H9c2 (H9c2 cells) and murine atrial cardiomyocytes (HL-1 cells) were purchased from American Type Culture Collection (ATCC). H9c2 and HL-1 cells were cultured in DMEM medium with 10% FBS, 1% penicillin and streptomycin antibiotics. Cells were incubated in an atmosphere of 5% CO2 at 37˚C. When the cells reached 50% confluency, H9c2 and HL-1 cells were pretreated with FG-4592 (5 μM) for 24 h and then stimulated with DOX (1 µM) for additional 24 h. After these treatments, cells were harvested for appropriate analyses.

### DOX-Sensitive Tumor Cell Studies

DOX-sensitive cell lines of HepG2 (liver hepatocellular carcinoma cells) and MCF-7 (human breast adenocarcinoma cell line) were pretreated with FG-4592 (5 μM) for 24 h and then stimulated with DOX (1 or 2 µM) for additional 24 h. After the treatments, cells were harvested for Cell viability assay and immunoblotting analyses.

### Cell Viability Assay

The viability of cells was measured by the CCK-8 assay kit. H9c2, HL-1, NRVM, HepG2, and MCF-7 cells were cultured in 96-well plate with DMEM medium for 24 h and then cells were treated with DOX (1-10 µM) for 24 h or FG-4592 (1–20 µM) for 48 h. Additionally, FG-4592 (5 µM) was used to pretreat cells for 24 h and then stimulated with DOX (1 or 2 µM) for another 24 h. After washing with PBS, cells were treated with 100 µl DMEM medium containing 10 µl CCK-8 solution for another 2 h at 37˚C. The values were measured by microplate reader at 450 nm (BioTek Instruments, Inc., Winooski, VT, United States).

### Quantitative Real-Time PCR (qRT-PCR)

Briefly, the total RNA from left ventricular tissues and H9c2 cells was extracted using TRIzol (TAKARA, Japan). The first-strand cDNA was synthesized from 1μg RNAs in a 10 µl reaction using reverse transcriptase (TAKARA, 2641A) following the manufacturer’s instructions. qRT-PCR was performed by using 7500 Fast PCR system (Applied Biosystems, Foster City, CA) with SYBR Mix (Applied Biosystems, Foster City, CA). Primers were listed in **Table 1**. The result of each sample was normalized to the expression of β-actin.

**Table 1:** Sequences of primers for qRT-PCR.

Gene name	Forward primer sequence (5’-3’)	Reverse primer sequence (5’-3’)
Mouse mt-ATP6	F: CCATAAATCTAAGTATAGCCATTCCAC	R: AGCTTTTTAGTTTGTGTCGGAAG
Mouse mt-ATP8	F: ACATTCCCACTGGCACC	R: GGGGTAATGAATGAGGC
Mouse mt-ND4L	F: GCCATCTACCTTCTTCA	R: TAGGGCTAGTCCTACAGC
Mouse mt-COX1	F: CAGACCGCAACCTAAACACA	R: TTCTGGGTGCCCAAAGAAT
Mouse mt-COX2	F: GCCGACTAAATCAAGCAACA	R: CAATGGGCATAAAGCTATGG
Mouse mt-COX3	F: CGTGAAGGAACCTACCAAGG	R: ATTCCTGTTGGAGGTCAGCA
Mouse mt-ND1	F: ACACTTATTACAACCCAAGAACACAT	R: TCATATTATGGCTATGGGTCAGG
Mouse mt-ND2	F: CCATCAACTCAATCTCACTTCTATG	R: GAATCCTGTTAGTGGTGGAAGG
Mouse mt-ND3	F: CCCCAAATAAATCTGTA	R: CTCATGGTAGTGGAAGT
Mouse mt-ND4	F: GCTTACGCCAAACAGAT	R: TAGGCAGAATAGGAGTGAT
Mouse mt-ND5	F: GCCAACAACATATTTCAACTTTTC	R: ACCATCATCCAATTAGTAGAAAGGA
Mouse mt-ND6	F: GGGAGATTGGTTGATGTA	R: ATACCCGCAAACAAAGAT
Mouse mt-CYTB	F: GAGGTTGGTTCGGTTTTGG	R: GTTTTGAAAGGGTGGGTGAC
Mouse β-actin	F: GAGACCTTCAACACCCCAGC	R: ATGTCACGCACGATTTCCC
Mouse Bcl-2	F: GCTACCGTCGTGACTTCGC	R: CCCCACCGAACTCAAAGAAGG
Mouse Bax	F: AGACAGGGGCCTTTTTGCTAC	R: AATTCGCCGGAGACACTCG
Mouse NIX	F: ATGTCTCACTTAGTCGAGCCG	R: CTCATGCTGTGCATCCAGGA
Rat TNF-α	F: GCGTGTTCATCCGTTCTCTACC	R: TACTTCAGCGTCTCGTGTGTTTCT
Rat IL-6	F: AGTTGCCTTCTTGGGACTGATGT	R: GGTCTGTTGTGGGTGGTATCCTC
Rat β-actin	F: AACCCTAAGGCCAACCGTG	R: TGCTCGAAGTCTAGGGCAAC

### Enzyme-Linked Immunosorbent Assay (ELISA)

The heart left ventricular and circulating TNF-α and IL-6 levels were measured by the ELISA kits following the manufacturer’s instructions.

### Immunoblotting

Immunoblotting was performed to detect the protein levels. Briefly, heart left ventricular tissues, cultured H9c2 and HepG2 cells were lysed with RIPA buﬀer with protease inhibitors cocktail for 30 min at 4°C. The cell lysates were centrifuged for 15 min (4°C and 12,000 g), and concentration of supernatants was confirmed by BCA method. After denatured, 50 μg proteins were loaded and separated by SDS-PAGE gel, and then transferred onto polyvinylidene difluoride (PVDF) membrane at 300 mA for 60 min. The PVDF membranes were blocked by 5% non-fat milk for 1 h and then incubated with primary antibodies (HIF-1α, 1:200; BAX, 1:1000; Bcl-2, 1:1000; SOD2, 1:1000; β-actin, 1:2000) at 4°C overnight. After incubating with secondary antibody for 2 h. The blots were detected with the enhanced chemiluminescence detection system (Bio-Rad, Hercules, CA, UK).

### Cell Apoptosis Analysis

After treatments, cells were harvested for double staining with FITC-annexin V and PI or FITC-annexin V and 7-AAD by using apoptosis detection kit according to the instruction. The cell apoptosis was detected with a flow cytometer (Beckman coulter, CytoFLEX) and the results were analyzed with CytExpert software.

### TUNEL Staining Analysis

Briefly, mice left ventricular tissues and H9c2 cells were fixed in 4% PFA for 30 min at room temperature and permeabilized with 0.1% Triton X-100 for 5 min. Subsequently, these tissues and cells were incubated with 50 μl of terminal deoxynucleotidyl transferase (TdT) Reaction mixture for 60 min at 37°C. After the incubation, 5 μg/ml DAPI was applied for nuclear staining for 10 min. The apoptotic cells were detected by fluorescence microscope and the rate of apoptosis was shown as the percentage of the number of condensed TUNEL positive nuclei to the number of DAPI stained nuclei under 200 x magniﬁcation ﬁeld.

### Statistical Analysis

All data were shown as the means ± SE. GraphPad Prism software was used as the statistical analyses tool. Data were analyzed by analysis of variance (ANOVA) followed by *post hoc* t test or Student’s t test. P values < 0.05 were considered the significant difference statistically.

## Results

### FG-4592 Pretreatment Alleviated DOX-Induced Acute Cardiac Dysfunction in Mice

**Figure 1:**
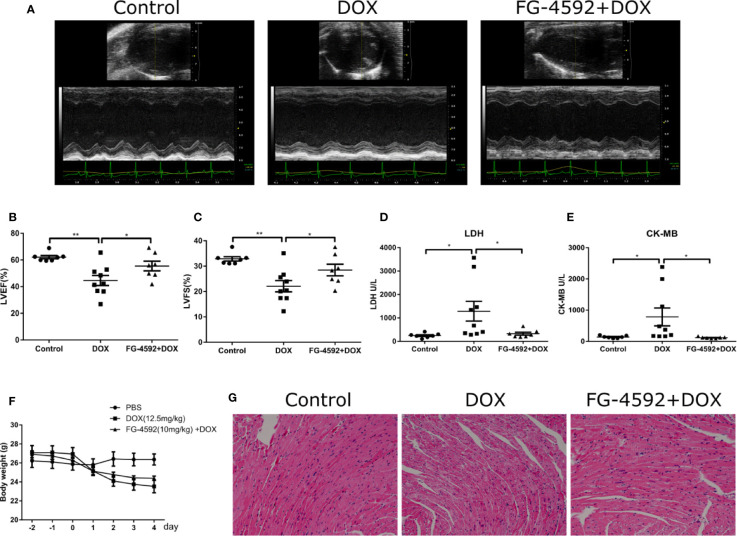
FG-4592 attenuated DOX-induced acute cardiotoxicity. **(A)** Representative images of echocardiography from experimental animals. **(B)** LVEF, left ventricular ejection fraction. **(C)** LVFS, left ventricular fractional shortening. **(D)** Serum concentrations of LDH. **(E)** Serum concentration of CK-MB. **(F)** Body weight of experimental animals. **(G)** Representative H&E staining images from the left ventricular of experimental animals. The values were represented as mean ± SED (n = 7–9 per group). *P < 0.05 and **P < 0.01.

To evaluate the effects of FG-4592 on the cardiac function in the DOX-induced acute cardiac dysfunction mice, we pretreated mice with FG-4592 at a dose of 10 mg/kg for 48 h, followed by a single injection of DOX (12.5 mg/kg). After DOX administration for 7 days, the cardiac function was assessed by echocardiography (**Figure 1A**). As expected, FG-4592 rescued the reduction of LVEF and LVFS induced by DOX (**Figures 1B, C**). LDH and CK-MB are known biomarkers for clinical diagnosis of cardiotoxicity. Compared to the control mice, LDH and CK-MB levels were significantly higher in DOX-treated animals (**Figures 1D, E**), which was remarkably blunted by FG-4592 treatment with no obvious effect on body weight lowering (**Figure 1F**). Furthermore, HE staining was performed to evaluate the cardiomyocyte morphology. In agreement with the improved cardiac function, FG-4592 alleviated cytoplasmic vacuolization of cardiomyocytes induced by DOX (**Figure 1G**). All these data indicated that FG-4592 obviously attenuated DOX-induced acute cardiac dysfunction and cardiomyocyte injury.

### FG-4592 Blocked the Apoptosis of Cardiomyocytes in DOX-Treated Mice

**Figure 2:**
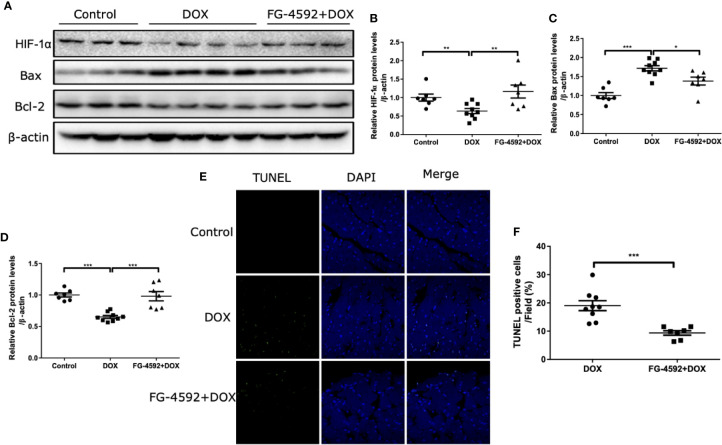
FG-4592 attenuated DOX-induced cardiomyocyte apoptosis. **(A)** The protein levels of HIF-1α, Bax, and Bcl-2 in the left ventricular from experimental animals were measured by immunoblotting analysis. β-actin was used as the loading control. **(B–D)** Quantitation of the Western blots of HIF-1α, Bax, and Bcl-2. **(E)** TUNEL staining of the left ventricular tissues from experimental animals. Nuclei were shown in blue, and TUNEL staining was shown in green. **(F)** Proportion of TUNEL-positive cells. The values were represented as mean ± SED (n = 7-9 per group). *P < 0.05, **P < 0.01, and ***P < 0.001.

Apoptosis is a known pathological phenomenon in DOX-induced acute cardiac injury. Thus, we detected the role of FG-4592 on apoptosis-associated proteins including Bax and Bcl-2 by immunoblotting and found that FG-4592 reversed the upregulation of Bax and the downregulation of Bcl-2 in response to DOX treatment (**Figures 2A–D**). Furthermore, TUNEL assay was performed in the left ventricular tissue of heart. As shown in **Figures 2E, F**, TUNEL-positive cell number in FG-4592+DOX group was less than the number in DOX alone group significantly. All these results suggested that FG-4592 could ameliorate apoptotic response in the heart of DOX-treated mice.

### FG-4592 Attenuated Mitochondrial Oxidative Stress in DOX-Treated Mice

**Figure 3:**
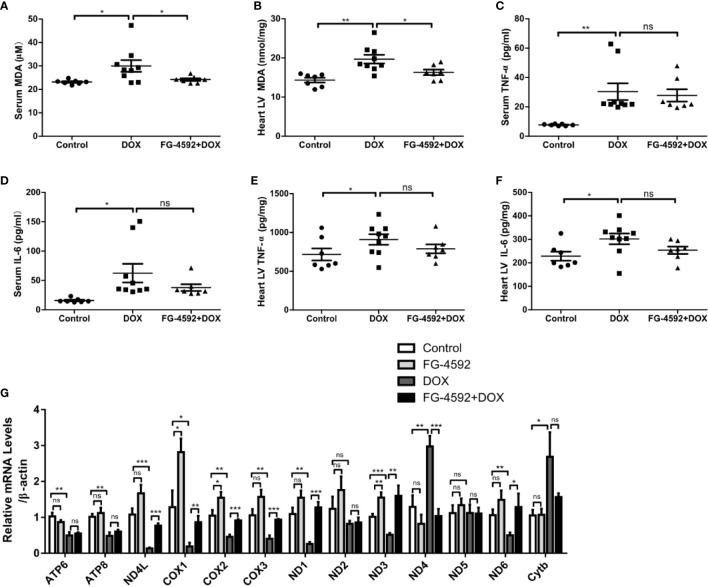
FG-4592 attenuated DOX-induced mitochondrial oxidative stress with no effect on inflammation. **(A)** Serum MDA level of the experimental animals. **(B)** MDA content in the left ventricular from experimental animals. **(C)** Serum TNF-α level in the experimental animals. **(D)** Serum IL-6 level in the experimental animals. **(E)** TNF-α level in the left ventricular from experimental animals. **(F)** IL-6 level in the left ventricular from experimental animals (n = 7–9 per group). **(G)** mRNA levels of 13 mitochondria-encoded genes in the left ventricular tissues from experimental animals by qRT-PCR (n=6 per group). The values were represented as mean ± SED. *P < 0.05, **P < 0.01, and ***P < 0.001.

**Figure 4:**
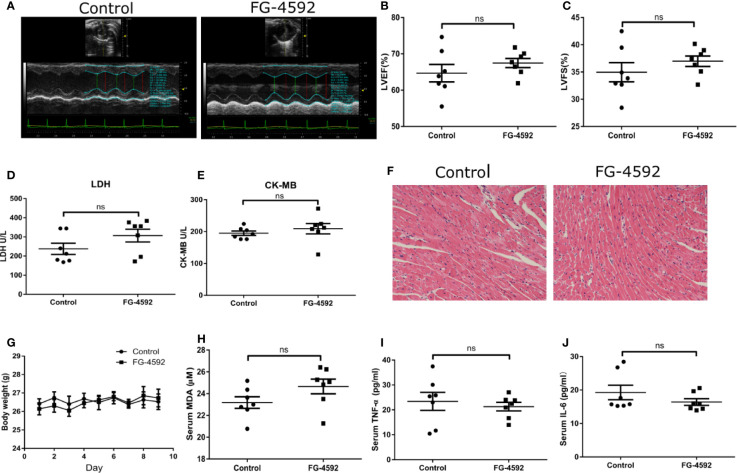
Assessment of cardiotoxicity of FG-4592 in mice. **(A)** Representative images of echocardiography from control group and FG-4592 group. **(B)** LVEF, left ventricular ejection fraction. **(C)** LVFS, left ventricular fractional shortening. **(D)** Serum concentration of LDH. **(E)** Serum concentration of CK-MB. **(F)** Representative H&E staining images from the left ventricular tissues of experimental animals (200×magnification). **(G)** Body weight of control group and FG-4592 group. **(H)** Serum MDA level between groups. **(I)** Serum TNF-α level of the two group; **(J)** Serum IL-6 level of the two groups; The values were represented as mean ± SED (n =7 per group).

Accumulating evidence has shown that excessive oxidative stress plays a critical role in the process of acute cardiac dysfunction induced by DOX. Malondialdehyde (MDA), an oxidative stress indicator, was measured in serum and heart left ventricular tissue. As expected, FG-4592 significantly reversed the enhanced MDA levels in serum and heart induced by DOX (**Figures 3A, B**). Besides oxidative stress, inflammation also play a role in DOX cardiotoxicity. Therefore, we measured the levels of TNF-α and IL-6 in serum and heart tissue by ELISA. Unexpectedly, DOX-induced increments of TNF-α and IL-6 in serum and heart were unable to be affected by FG-4592 treatment (**Figures 3C–F**). Furthermore, we analyzed the mitochondrial status by detecting the mRNA levels of 13 mitochondrial-encoded genes. As shown in **Figure 3G**, FG-4592 could reverse the dysregulation of most mitochondrial-encoded genes including ND4L, COX1, COX2, COX3, ND1, ND3, ND4, and ND6 regulated by DOX. These results indicated that FG-4592 protected heart against DOX challenge possibly by protecting mitochondria and reducing mitochondrial oxidative stress without the effect on modulating inflammation. To assess the potential toxicity of FG-4592 on heart, we performed another animal experiment including both control and FG-4592 groups. As shown by the data (**Figure 4**), FG-4592 had no obvious side effect on heart function and morphology.

### FG-4592 Activated HIF-1α and Its Target Genes of Bcl-2 and SOD2

**Figure 5:**
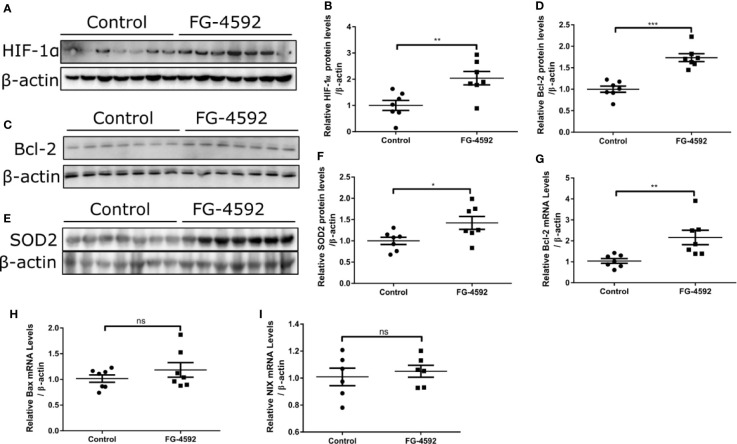
FG-4592 enhanced cardiac expression of HIF-1α, SOD2, and Bcl-2 in mice. **(A, C, E)** Western blots of HIF-1α, Bcl-2, and SOD2 in the left ventricular tissues from control and FG-4592-treated mice were measured by immunoblotting analysis. β-actin was used as the loading control. **(B)** Quantitation of the Western blots of HIF-1α. **(D)** Quantitation of the Western blots of Bcl-2. **(F)** Quantitation of the Western blots of SOD2. **(G–I)** mRNA levels of Bcl-2, BAX and NIX in the left ventricular tissues from control and FG-4592-treated mice by qRT-PCR. n=6-7 per group. *P < 0.05, and **P < 0.01.

In order to assess the role of FG-4592 in HIF-α activation, we performed Western blotting to analyze the expression of HIF-1α. As shown in **Figures 5A, B**, FG-4592 treatment upregulated the protein level of HIF-1α in heart. Furthermore, we examined HIF target genes including BAX, NIX, Bcl-2, and SOD2 and found that both the protein and mRNA levels of Bcl-2 and SOD-2 were up-regulated in FG-4592 treatment group without affecting the expression of BAX and NIX (**Figures 5C–I**). These results indicated that FG-4592 could activate HIF-1α and increase its target genes of Bcl-2 (an anti-apoptotic protein) and SOD2 (an antioxidant enzyme) to protect heart against injury.

### FG-4592 Prevented DOX-Induced Cell Apoptosis *In Vitro*

**Figure 6:**
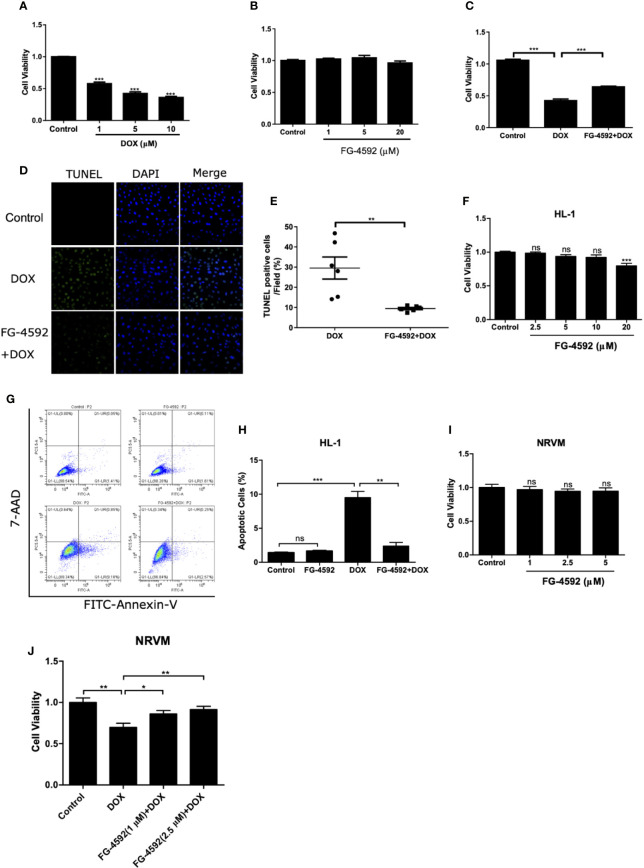
FG-4592 prevented DOX-induced cell apoptosis in cardiac cells. **(A)** Cell viability was detected by CCK-8 assay kit in H9c2 cells with the treatment of DOX at the concentrations from 1 µM to 10 µM for 24 h. **(B)** Cell viability was detected by CCK-8 assay kit in H9c2 cells with the treatment of FG-4592 at the concentrations from 1 µM to 20µM for 48 h. **(C)** H9c2 cells were pretreated with FG-4592 (5 μM) for 24 h followed by DOX (1 μM) treatment for another 24 h. Cell viability was detected by CCK-8 assay (n =6 per group). **(D)** Representative images of TUNEL staining. Nuclei were shown in blue, and TUNEL staining was shown in green. **(E)** Proportion of TUNEL-positive cells (n =6 per group). **(F)** Cell viability was detected by CCK-8 assay kit in HL-1 cells with the treatment of FG-4592 at the concentrations from 2.5 µM to 20µM for 48 h (n =6 per group). **(G)** Representative images of FACS analysis for cell apoptosis. HL-1 cells were pretreated with FG-4592 (5 μM) for 24 h followed by DOX (1 μM) treatment for another 24 h. **(H)** Quantification of the percentage of apoptotic cells (n =3 per group). **(I)** Cell viability was detected by CCK-8 assay kit in NRVM cells with the treatment of FG-4592 at the concentrations from 1 µM to 5µM for 48 h (n =6 per group). **(J)** NRVM cells were pretreated with FG-4592 (1 or 2.5 μM) for 24 h followed by DOX (1 μM) treatment for another 24 h. Cell viability was detected by CCK-8 assay (n =6 per group). The values were represented as mean ± SED. *P < 0.05, **P < 0.01, and ***P < 0.001.

**Figure 7:**
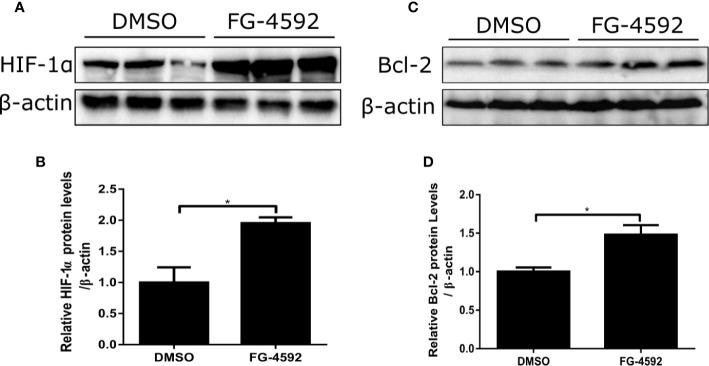
FG-4592 effect on regulating HIF-1α and its target gene Bcl-2 in DOX-treated H9c2 cells. H9c2 cells were treated with FG-4592 (5 μM) or DMSO for 48 h. **(A, C)** Expression of HIF-1α and Bcl-2 were examined by immunoblotting analysis. β-actin was used as the loading control. **(B, D)** Quantitation of the Western blots of HIF-1α and Bcl-2. The values were represented as mean ± SED (n =3 per group). *P < 0.05.

To further evaluate the direct effect of FG-4592 *in vitro*, we applied DOX to H9c2 cell line to induce cardiomyocyte injury. First, CCK-8 assay was performed to evaluate the cell toxicity of DOX and FG-4592 on H9c2 cells. DOX (1–10 µM) and FG-4592 (1–20 µM) were used to treat H9c2 cells for 24 h and 48 h, respectively. DOX inhibited H9c2 cells viability in a dose-dependent manner (**Figure 6A**). Significantly, 1 µM DOX was able to cause cell toxicity, which is consistent with the previous research report showing that the serum DOX concentration in patients with DOX chemotherapy was around 1µM (Frost et al., 2002). Therefore, 1 µM DOX was applied in subsequent experiments. FG-4592 treatment with the doses from 1 to 20 µM showed no obvious toxicity on H9c2 cells (**Figure 6B**). In DOX-treated H9c2 cells, FG-4592 at the dose of 5 µM significantly improved cell viability and cell apoptosis as detected by CCK-8 and TUNEL assays, respectively (**Figures 6C–E**). Meanwhile, we also evaluated the role of FG-4592 in HL-1 and NRVM cells treated with DOX and found that FG-4592 significantly improved cell apoptosis in HL-1 cells and cell viability in NRVMs (**Figures 6F–J**). Furthermore, we confirmed that FG-4592 could increase HIF-1α and Bcl-2 protein in H9c2 cells (**Figures 7A–D**). Above data demonstrated a direct effect of FG-4592 on protecting against DOX-induced cardiac cell apoptosis by targeting HIF-1α and Bcl-2.

### FG-4592 Prevented DOX-Induced Mitochondrial Oxidative Stress in H9c2 Cells

**Figure 8:**
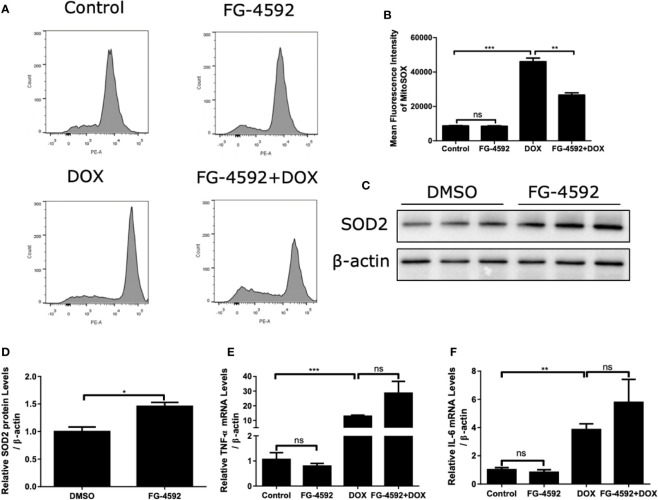
FG-4592 prevented DOX-induced mitochondrial oxidative stress in H9c2 cells. H9c2 cells were pretreated with FG-4592 (5 μM) for 24 h followed by DOX (1 μM) treatment for another 24 h. **(A)** MitoSOXTM Red Mitochondrial superoxide indicator was applied to analyze the production of intracellular mitochondrial ROS. Representative images of the flow cytometry data. **(B)** Quantitation of the mean fluorescence intensity. **(C)** H9c2 cells were treated with FG-4592 (5 μM) or DMSO for 48 h, expression of SOD2 was examined by immunoblotting analysis. β-actin was used as the loading control. **(D)** Quantitation of the Western blots of SOD2. **(E, F)** The mRNA level of TNF-α and IL-6 were measured by qRT-PCR. The values were represented as mean ± SED (n =3 per group). *P < 0.05, **P < 0.01, and ***P < 0.001.

Mitochondrial dysfunction and excessive ROS production are of importance in mediating cell injury induced by DOX. Thus, MitoSOX™ Red Mitochondrial superoxide indicator was used to analyze the intracellular mitochondrial ROS generation. As shown by the flow cytometry data in **Figures 8A, B**, FG-4592 significantly suppressed mitochondrial ROS generation induced by DOX treatment. In addition, FG-4592 could upregulate HIF-1α target gene SOD2 protein, a potent antioxidant enzyme (**Figures 8C, D**). Furthermore, the mRNA levels of TNF-α and IL-6 were confirmed by qRT-PCR in DOX-induced H9c2 cells. Similar as the *in vivo* results, FG-4592 did not ameliorate the inflammation response induced by DOX in H9c2 cells (**Figures 8E, F**). These results further indicated an antioxidant effect of FG-4592 against DOX challenge in H9c2 cells.

### FG-4592 Promoted the Anticancer Activity of DOX

**Figure 9:**
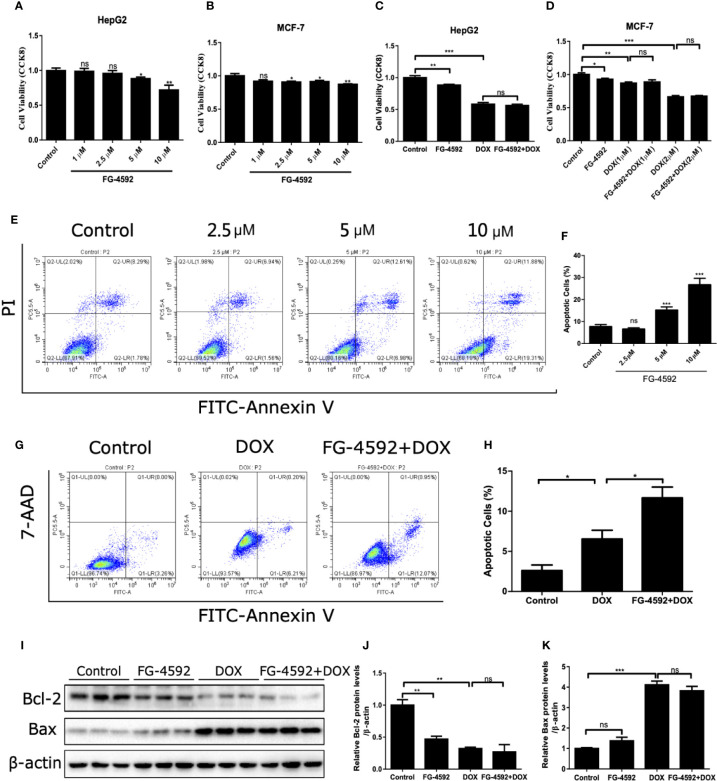
FG-4592 effect on the antitumor activity of DOX. **(A, B)** HepG2 and MCF-7 cells were treated with different dose of FG-4592(1, 2.5, 5, 10 μM) for 24 h, Cell viability was detected by CCK-8 assay (n =6 per group). **(C)** HepG2 cells were pretreated with FG-4592 (5 μM) for 24 h followed by DOX (1 μM) treatment for another 24 h. Cell viability was detected by CCK-8 assay (n =6 per group). **(D)** MCF-7 cells were pretreated with FG-4592 (5 μM) for 24 h followed by DOX (1 or 2 μM) treatment for another 24 h. Cell viability was detected by CCK-8 assay (n =6 per group). **(E)** Representative images of FACS analysis for cell apoptosis. HepG2 cells were treated with different doses of FG-4592 (2.5, 5, and 10 μM) for 48 h. **(F)** Quantification of the percentage of apoptotic cells (n =6 per group). **(G)** Representative images of FACS analysis for cell apoptosis. HepG2 cells were pretreated with FG-4592 (5 μM) for 24 h followed by DOX (1 μM) treatment for another 24 h. **(H)** Quantification of the percentage of apoptotic cells (n =3 per group). **(I)** Expression of Bcl-2 and Bax were examined by immunoblotting analysis. β-actin was used as the loading control. **(J, K)** Quantitation of the Western blots of Bcl-2 and Bax (n =3 per group). The values were represented as mean ± SED. *P < 0.05, **P < 0.01, and ***P < 0.001.

Finally, we assessed the effect of FG-4592 on anticancer activity of DOX. As shown by the data, FG-4592 alone could inhibit the tumor cell viability but had no further effect on the reduced cell viability in HepG2 and MCF-7 cells treated with DOX (**Figures 9A–D**). Moreover, the results shown in **Figures 9E–H** showed that FG-4592 could aggravate the apoptosis of HepG2 cells with or without DOX treatment. Consistently, FG-4592 reduced antiapoptotic protein Bcl-2 in HepG2 cells (**Figures 9I–K**), which is different from the findings in cardiac cells. Taken together, these results suggested that the combined use of FG-4592 and DOX might strengthen the antitumor effect of DOX.

## Discussion

As a chemotherapy drug broadly used in patients with various cancers, DOX has a recognized therapeutic effect on cancers. However, the cardiotoxicity of DOX limited its clinical administration. Nowadays, dexrazoxane is the only cardio-protective drug against DOX cardiotoxicity (QuanJun et al., 2017). However, dexrazoxane was found to interfere with the antitumor effect of DOX (Imondi, 1998). Therefore, it is necessary to find a protective strategy against the cardiotoxicity of DOX without disturbing its anti-cancer effect. A recently study reported that FG-4592 could enhance erythropoiesis with no interference on the initiation, progression, or metastasis of cancer (Seeley et al., 2017). In this work, we investigated the effect of FG-4592 on DOX-induced acute myocardial injury. FG-4592 is a new HIF-PHD inhibitor that has been approved in China to treat CKD anemia by stabilizing HIF-1α. HIF-1α is an important transcription factor with many target genes which participate in angiogenesis, erythropoiesis, apoptosis and energy metabolism (Carmeliet et al., 1998; Vincent et al., 2000; Kakinuma et al., 2001; Liu et al., 2003). HIF-1α has been identified to play a protective role in the ischemic heart disease, atherosclerosis, and heart failure. Activation of HIF-1α improves the tolerance to ischemia and hypoxia in myocardial cells and reduces the apoptosis of myocardial cells by regulating glycolysis and energy metabolism (Eckle et al., 2012; Ong et al., 2014). Another study showed HIF-1α blunted cell injury by suppressing apoptosis and oxidative stress in H9c2 cells (Spagnuolo et al., 2011). In agreement with this study, our results suggested that FG-4592 could protect against DOX-induced myocardial toxicity possibly by improving apoptosis and mitochondrial oxidative stress, and the protective effect of FG-4592 might be associated with the stabilization of HIF-1α.

Apoptosis is documented as a common phenomenon of myocardial toxicity induced by DOX (Kalyanaraman et al., 2002). Previous study found that FG-4592 pretreatment could reduce cisplatin-induced renal tubular cell apoptosis by stabilizing the expression of HIF (Yang et al., 2018). Another *in vitro* study demonstrated HIF played a critical role in mediating the myocardial protective effect of dexrazoxane by up-regulating its target genes including survivin, Mcl-1 (an anti-apoptotic protein belonged to Bcl-2 gene family) and HO-1, which are all the anti-apoptotic proteins (Spagnuolo et al., 2011). Consistent with these findings, in the present study, FG-4592 treatment upregulated Bcl-2 expression, attenuated DOX-induced cardiomyocyte apoptosis, and protected heart function in response to DOX challenge.

Mitochondria are important source of ATP production in cardiomyocytes. Mitochondrial oxidative stress also causes mtDNA damage. mtDNA encodes 13 respiratory chain complex proteins playing key role for the maintenance of normal mitochondrial function (Ren et al., 2010). In agreement with these known concepts, we found DOX affected the expression of 13 mitochondrial genes, which was remarkably restored by FG-4592 pretreatment, indicating a potent role of FG-4592 in protecting mitochondria. Excessive mitochondrial ROS plays a key role in the pathogenesis of myocardial injury. When the ROS generation exceeds the scavenging capacity, the oxidative stress will be triggered, which will further cause severe damage to cardiomyocytes. Accumulating reports suggested that DOX could increase the levels of ROS and MDA, and decrease the activity of antioxidant enzymes like SOD2, GSH-PX and CAT in myocardial tissue. Consistent with these notions, we found that DOX significantly enhanced MDA levels in mouse serum and left ventricular tissue, and ROS level in H9c2 cells. After FG-4592 treatment, the oxidative stress was markedly blunted in DOX-treated mice and cardiomyocytes. As a HIF target gene, SOD2 protein level was significantly upregulated by FG-4592, which may contribute to the antioxidant effect of FG-4592 in this experimental setting.

Besides the apoptosis and oxidative stress, inflammatory response is also involved in the pathogenesis of DOX-induced cardiomyopathy. Several studies found that DOX induced the release of inflammatory factors of TNF-α and IL-6 in cardiac tissue (Akolkar et al., 2017; Yuan et al., 2018). In the present study, we also found an increment of inflammatory cytokines of TNF-α and IL-6 in cardiac tissue and H9c2 cells treated with DOX. However, FG-4592 pretreatment showed no effect on lowering the expression levels of TNF-α and IL-6. These results suggested that FG-4592 protected against DOX-induced cardiac injury independently of the anti-inflammatory action. In agreement with this notion, one research showed that HIF-1 could exacerbate colitis by promoting inflammatory cell infiltration (Shah et al., 2008), and the deletion of HIF-1α in macrophages reduced mortality induced by LPS (Peyssonnaux et al., 2007). Additionally, a recent study showed that FG-4592 could alleviate inflammation in cisplatin-induced acute kidney disease (Yang et al., 2018). The discrepancy of FG-4592 in modulating inflammation could be due to the diversity of HIF-1α function under different pathological conditions.

FG-4592 was found to have no interference on the initiation, progression, or metastasis of cancer (Seeley et al., 2017). However, recent studies showed an antitumor action of FG-4592 in macrophage-abundant tumors (Nishide et al., 2019), and in human ovarian clear cell carcinoma ES2 cells (Price et al., 2019). In another study, FG-4592 could selectively protect the intestinal tract from radiation toxicity without affecting pancreatic cancer (Fujimoto et al., 2019). In our present study, we treated DOX-sensitive cells of HepG2 and MCF-7 with FG-4592 in combination with DOX for 24 h and found FG-4592 had no further effect on the reduced cell viability induced by DOX. Interestingly, FG-4592 alone even inhibited tumor cell growth and induced tumor cell apoptosis. Furthermore, we observed that FG-4592 aggravated the apoptosis of HepG2 cells induced by DOX. The diverse action of FG-4592 in regulating cell apoptosis in cardiac cells and tumor cells could be due to the diversity of cell types or off-target action of this drug. All above findings indicated that FG-4592 could not antagonize the DOX effect on killing tumor cells.

In conclusion, we reported a potent protective effect of FG-4592 against DOX-induced cardiotoxicity in mice possibly through inhibiting apoptosis and oxidative stress. The findings also suggested that FG-4592 might be a promising drug for preventing DOX-induced cardiotoxicity besides its role in treating CKD anemia.

## Data Availability Statement

The raw data supporting the conclusions of this article will be made available by the authors, without undue reservation.

## Ethics Statement

The animal study was reviewed and approved by Institutional Animal Care and Use Committee of Nanjing Medical University.

## Author Contributions 

ZJ, WX, and HC designed the experiments, analyzed data, prepared the figures and wrote the manuscript. WX, GL, MW, and YL performed the experiments. LG, YZ, and SH contributed to technical advices. All authors reviewed the manuscript.

## Funding

The study was supported by the following funds: grants from the National Natural Science Foundation of China (No. 81600352, 81873599, 81830020, and 81670678); grant from Natural Science Foundation of Jiangsu Province (No. BK20160137); grant of China Postdoctoral Science Foundation (No. 2018M640504); grant of Postdoctoral Fund of Jiangsu Province (No. 2018K255C), grants from Nanjing National Commission on Health and Family Planning (No. YKK16183 and YKK18146), and the grant from Nanjing Science and Technology Commission (201823013).

## Conflict of Interest

The authors declare that the research was conducted in the absence of any commercial or financial relationships that could be construed as a potential conflict of interest.

## Supplementary Material

The Supplementary Material for this article can be found online at: https://www.frontiersin.org/articles/10.3389/fphar.2020.01191/full#supplementary-material
