# Supplementary material for: Antianemia Drug Roxadustat (FG-4592) Protects Against Doxorubicin-Induced Cardiotoxicity by Targeting Antiapoptotic and Antioxidative Pathways
Source: Front Pharmacol. 2020 Aug 5;11:1191. doi: 10.3389/fphar.2020.01191 (PMC7419679; doi:10.3389/fphar.2020.01191)
Supplement: Supplementary file 1 [file Presentation_1.pptx]

## Slide 1
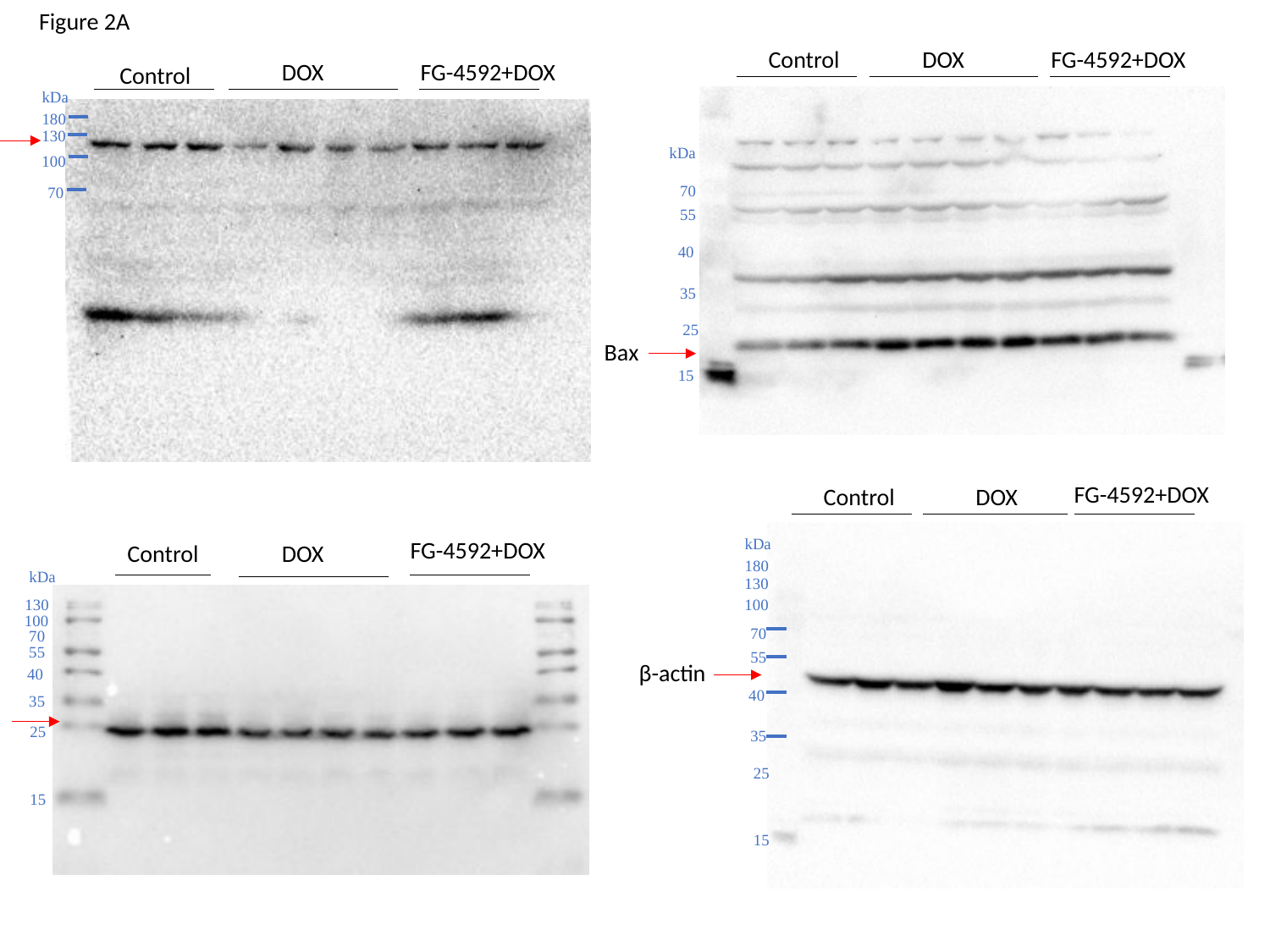

Figure 2A
Control
DOX
FG-4592+DOX
DOX
FG-4592+DOX
Control
kDa
180
130
100
70
HIF-1α
kDa
70
55
40
35
25
15
Bax
FG-4592+DOX
Control
DOX
kDa
180
130
100
70
55
40
35
25
15
FG-4592+DOX
Control
DOX
kDa
130
100
70
55
40
35
25
15
β-actin
Bcl-2

## Slide 2
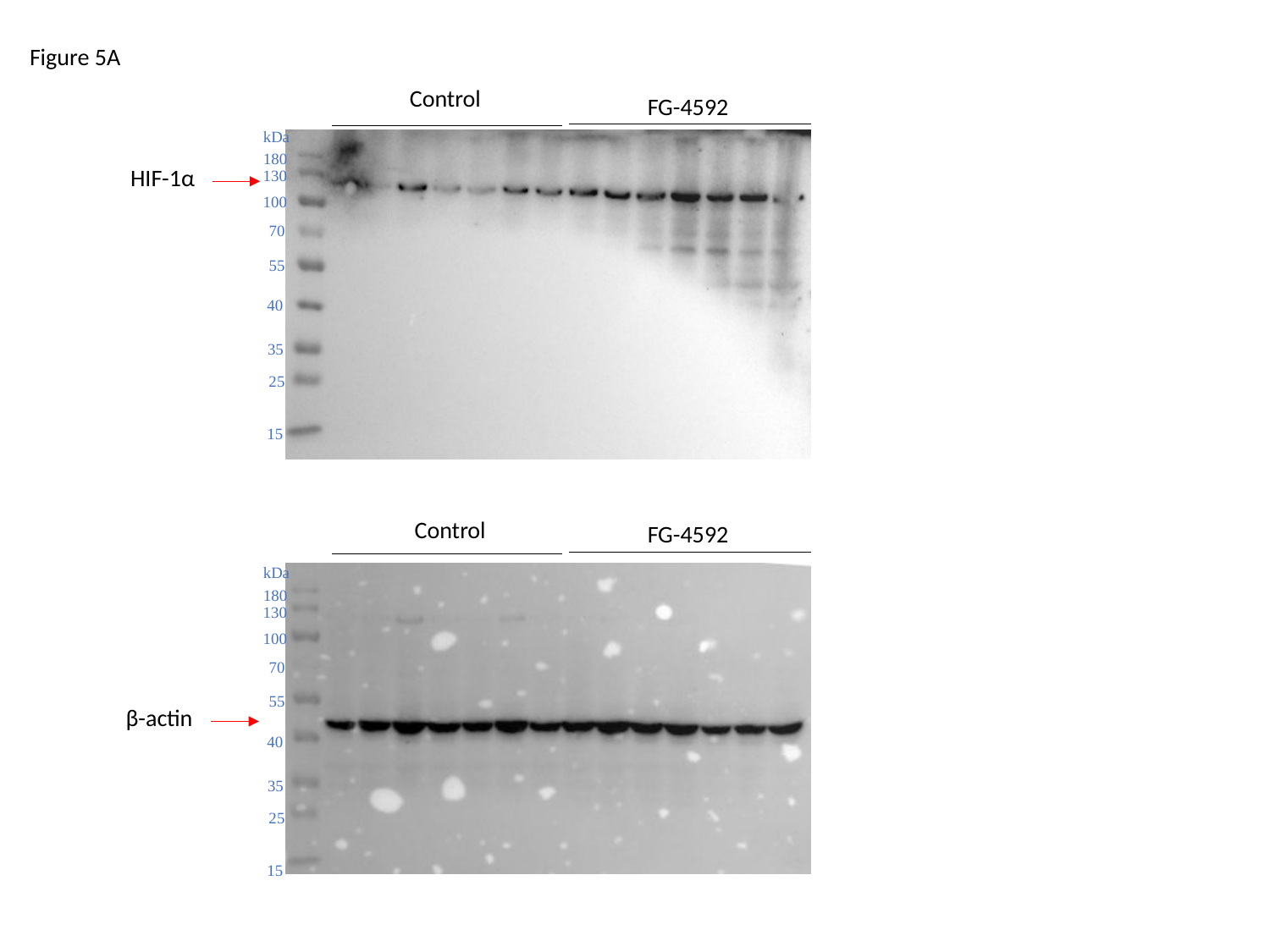

Figure 5A
Control
FG-4592
kDa
180
130
100
70
55
40
35
25
15
HIF-1α
Control
FG-4592
kDa
180
130
100
70
55
40
35
25
15
β-actin

## Slide 3
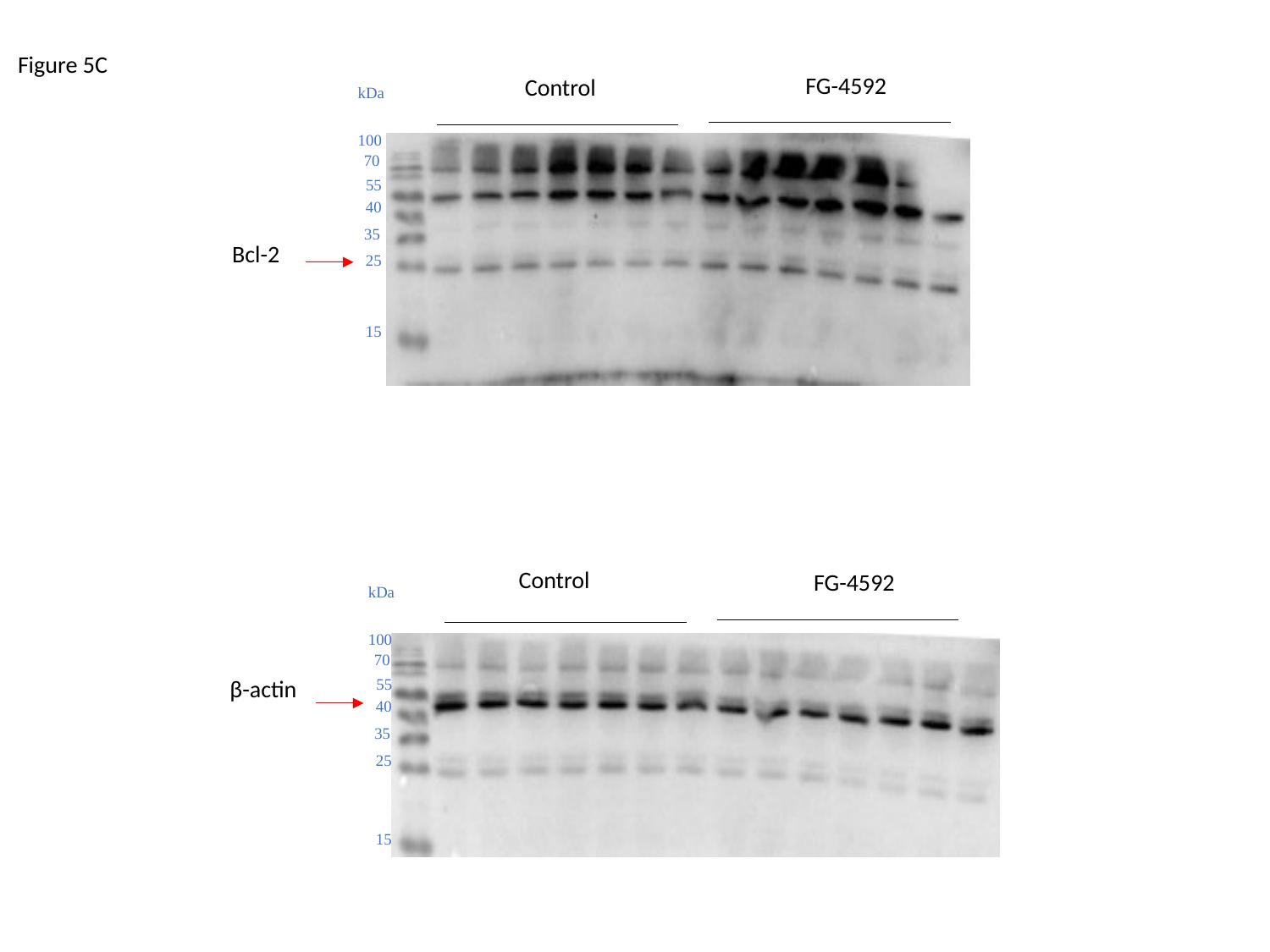

Figure 5C
FG-4592
Control
kDa
100
70
55
40
35
25
15
Bcl-2
Control
FG-4592
kDa
100
70
55
40
35
25
15
β-actin

## Slide 4
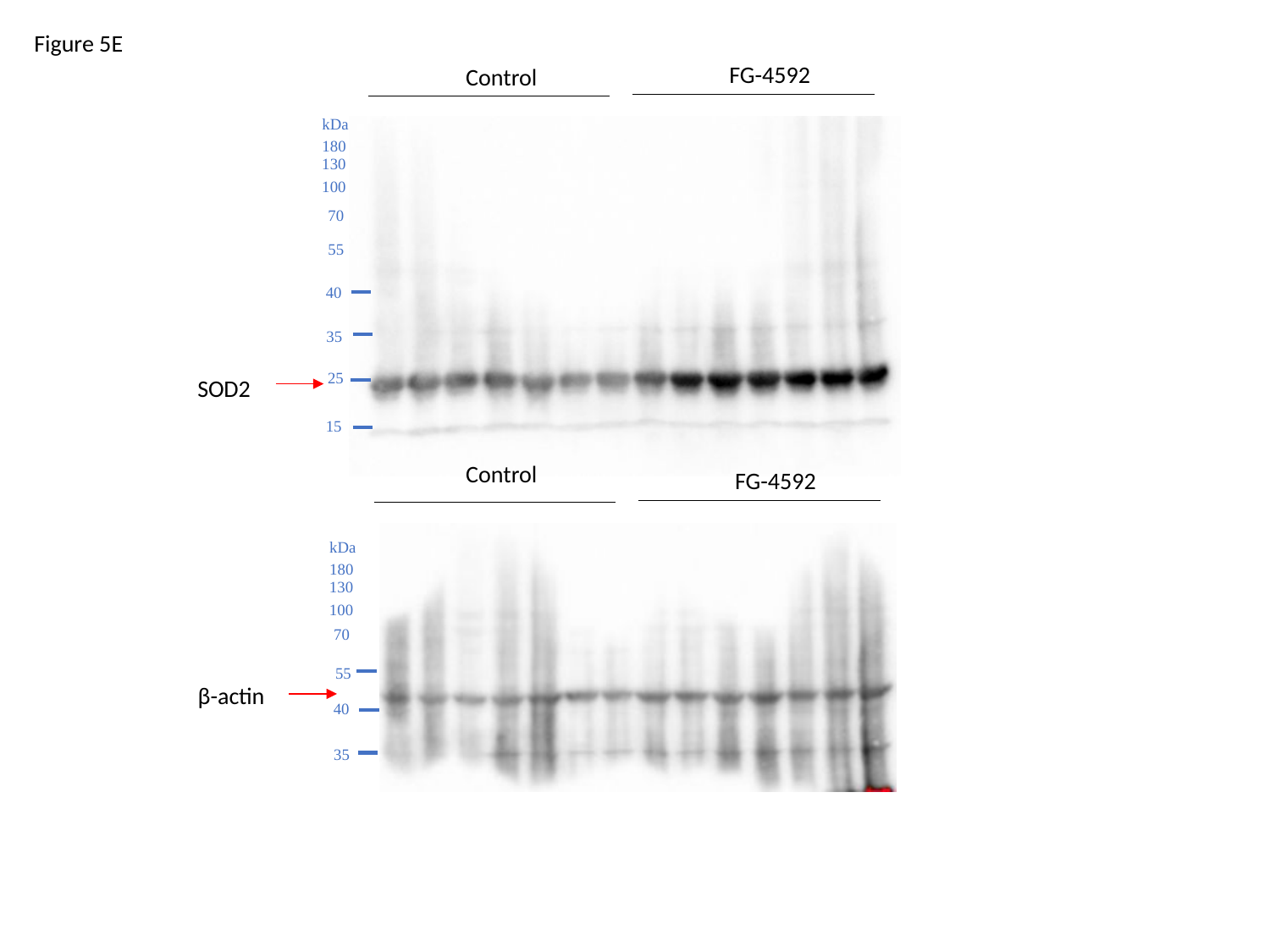

Figure 5E
FG-4592
Control
kDa
180
130
100
70
55
40
35
25
15
SOD2
Control
FG-4592
kDa
180
130
100
70
55
40
35
β-actin

## Slide 5
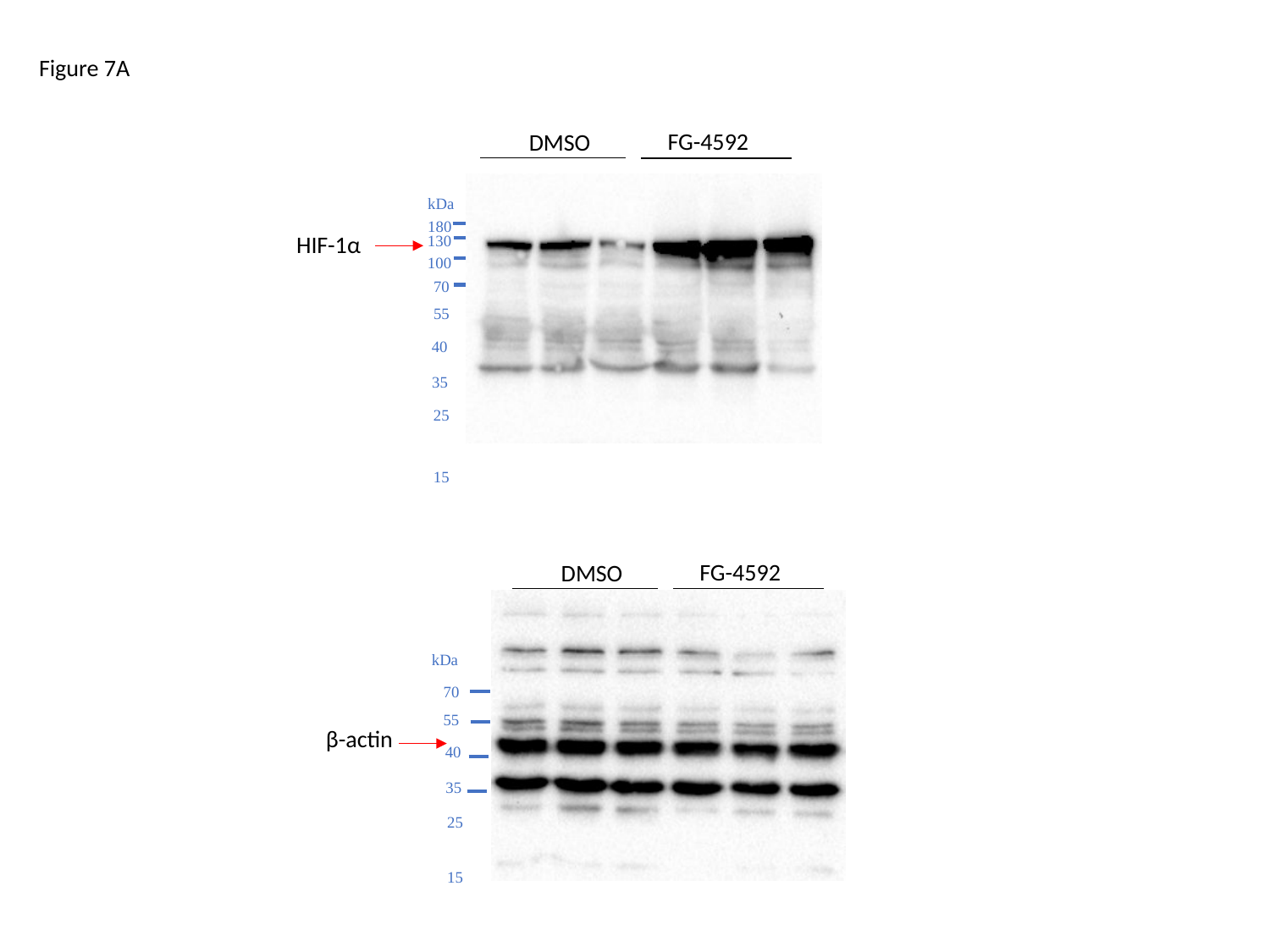

Figure 7A
FG-4592
DMSO
kDa
180
130
100
70
55
40
35
25
15
HIF-1α
FG-4592
DMSO
kDa
70
55
40
35
25
15
β-actin

## Slide 6
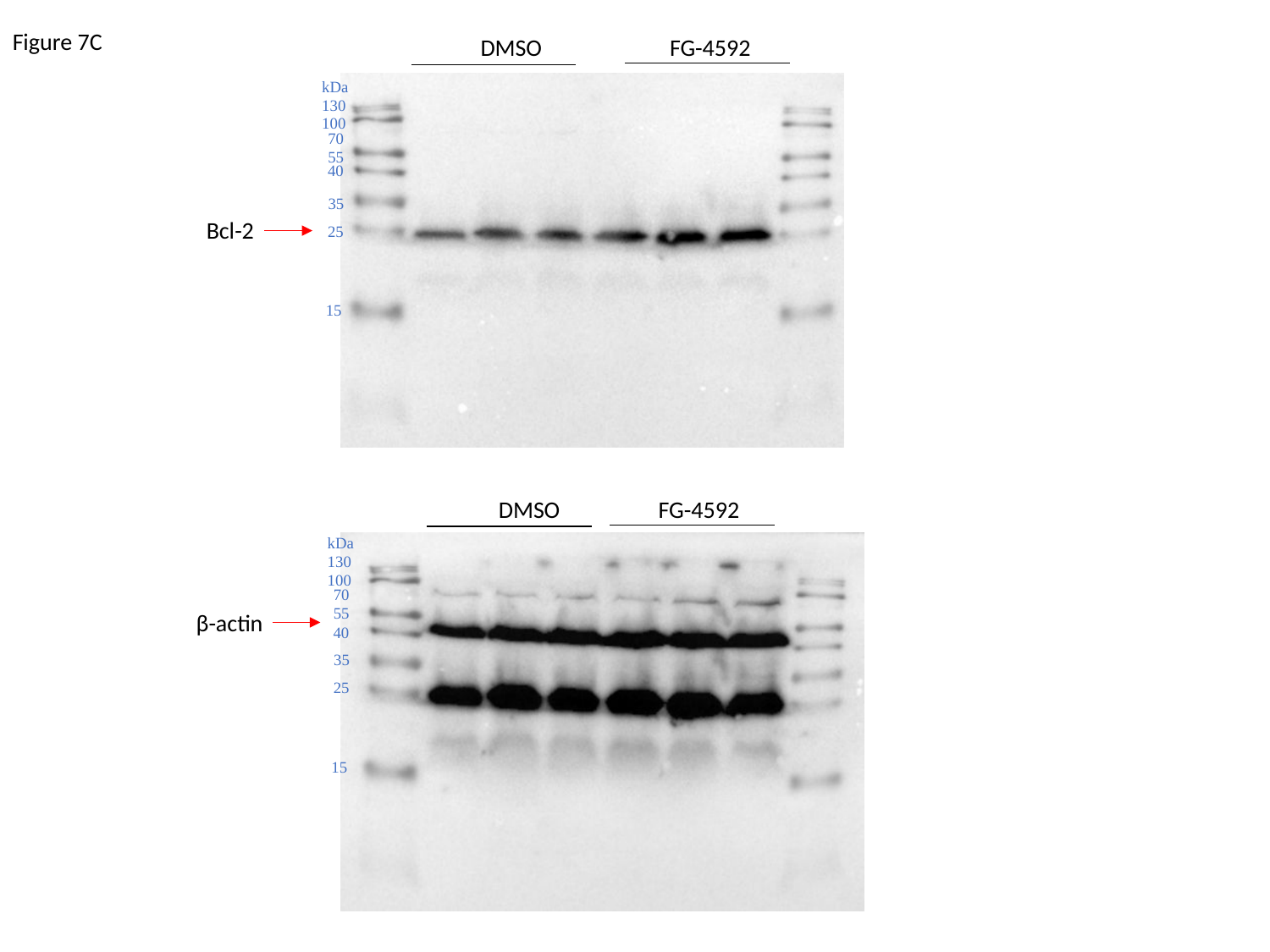

Figure 7C
DMSO
FG-4592
kDa
130
100
70
55
40
35
25
15
Bcl-2
DMSO
FG-4592
kDa
130
100
70
55
40
35
25
15
β-actin

## Slide 7
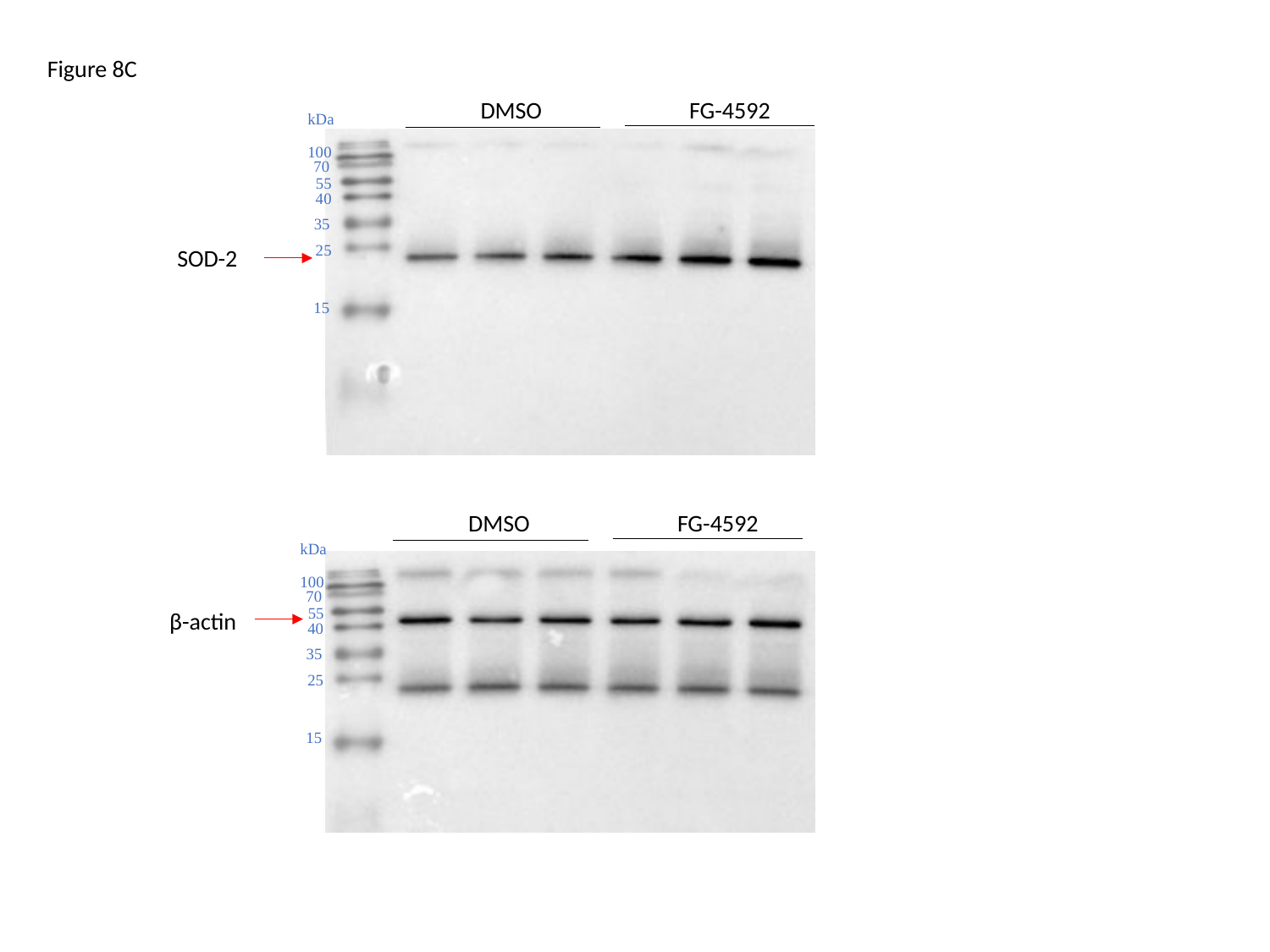

Figure 8C
DMSO
FG-4592
kDa
100
70
55
40
35
25
15
SOD-2
DMSO
FG-4592
kDa
100
70
55
40
35
25
15
β-actin

## Slide 8
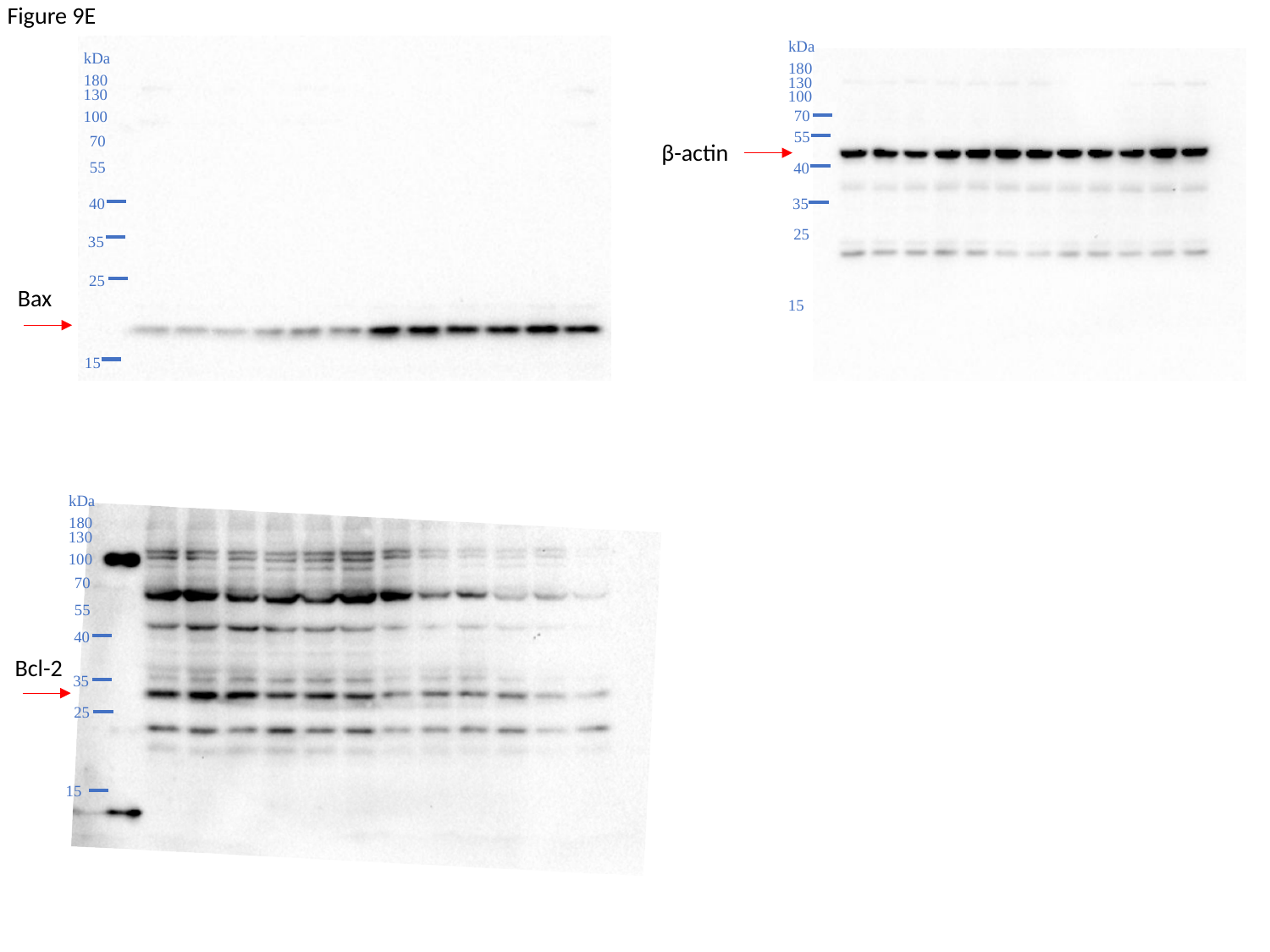

Figure 9E
kDa
180
130
100
70
55
40
35
25
15
kDa
180
130
100
70
55
40
35
25
15
β-actin
Bax
kDa
180
130
100
70
55
40
35
25
15
Bcl-2
